# The bachelor’s to Ph.D. STEM pipeline no longer leaks more women than men: a 30-year analysis

**DOI:** 10.3389/fpsyg.2015.00037

**Published:** 2015-02-17

**Authors:** David I. Miller, Jonathan Wai

**Affiliations:** ^1^Department of Psychology, Northwestern UniversityEvanston, IL, USA; ^2^Talent Identification Program, Duke UniversityDurham, NC, USA

**Keywords:** doctoral education, STEM education, gender differences, STEM persistence, retrospective methods

## Abstract

For decades, research and public discourse about gender and science have often assumed that women are more likely than men to “leak” from the science pipeline at multiple points after entering college. We used retrospective longitudinal methods to investigate how accurately this “leaky pipeline” metaphor has described the bachelor’s to Ph.D. transition in science, technology, engineering, and mathematics (STEM) fields in the U.S. since the 1970s. Among STEM bachelor’s degree earners in the 1970s and 1980s, women were less likely than men to later earn a STEM Ph.D. However, this gender difference closed in the 1990s. Qualitatively similar trends were found across STEM disciplines. The leaky pipeline metaphor therefore partially explains historical gender differences in the U.S., but no longer describes current gender differences in the bachelor’s to Ph.D. transition in STEM. The results help constrain theories about women’s underrepresentation in STEM. Overall, these results point to the need to understand gender differences at the bachelor’s level and below to understand women’s representation in STEM at the Ph.D. level and above. Consistent with trends at the bachelor’s level, women’s representation at the Ph.D. level has been recently declining for the first time in over 40 years.

## INTRODUCTION

For three decades, research and public discourse about gender differences in academic science have often focused on the “leaky pipeline” metaphor (Berryman, 1983; Alper, 1993). According to this metaphor, women are more likely than men to leave science at multiple time points from the beginning of college through academic tenure. Scholars from diverse fields have proposed how specific factors such as cognitive abilities, discrimination, and interests can explain these gender differences in opting out (Ceci et al., 2009). These interlocking factors could collectively cause “leaks” at various segments in the science pipeline and therefore lead to an underrepresentation of women among science Ph.D. holders and faculty. In this way, the leaky pipeline metaphor has explicitly and implicitly served as a core theoretical foundation for several explanations regarding the underrepresentation of women in science, technology, engineering, and mathematics (STEM) fields.

We investigated how accurately the leaky pipeline metaphor has described the bachelor’s to Ph.D. STEM pipeline in the U.S. since the 1970s. During this time frame, women’s representation in STEM fields has dramatically increased. For instance, women earned 19% of the U.S.’s bachelor’s degrees in chemistry in 1966, but earned 48% of them in 2013^1^. The increase in women’s representation at the Ph.D. and assistant professorship levels has also been dramatic (Ceci et al., 2014). Given this rapid change over time, it is especially worth considering whether the leaky pipeline metaphor (1) was empirically supported in the past, and (2) continues to be empirically supported today. Current inaccuracies in this metaphor could constrain and potentially prompt revision of diverse theories about current gender differences in STEM fields. Improving such conceptual models could also help policy makers target when and where to allocate limited resources for increasing gender diversity in STEM fields.

Recent research has found some current inadequacies of the leaky pipeline metaphor (Cannady et al., 2014; Miller et al., under review). For instance, plugging leaks in the pipeline from the beginning of college to the bachelor’s degree would fail to substantially increase women’s representation among U.S. undergraduates in physical science, technology, engineering, and mathematics (pSTEM) fields^2^ (excluding life science and social science). Women currently earn 25% of pSTEM bachelor’s degrees in the U.S., and equalizing gender differences in undergraduate pSTEM retention would only increase this percentage to 27% (Miller et al., under review).

Other research has found large gender differences in opting out exist only in some STEM fields, but not others. For instance, the percentage of women among academic biologists substantially declines from receiving a biology Ph.D. to applying for tenure-track positions at Research I institutions; this decline suggests a leaky academic pipeline for female biologists (National Research Council [NRC], 2010). However, such declines are counterintuitively far smaller in the more male-dominated fields of physics and engineering (National Research Council [NRC], 2010). This evidence and related studies have indicated that, when describing academic transitions after the Ph.D., the leaky pipeline metaphor is less accurate for the more male-dominated STEM fields – the fields for which the metaphor was originally intended (see Ceci et al., 2014 for a review).

We contribute to this research on persistence in STEM fields by investigating men’s and women’s transition from undergraduate to graduate education. During this formative period, students start to develop identities as scientists and engineers capable of independently producing scientific knowledge and technological innovations (Herzig, 2004). Several scholars have suggested that women face more challenges than men in completing this transition. Such challenges could include gender discrimination from academic mentors (Moss-Racusin et al., 2012; Milkman et al., 2014), male advantages on gatekeeper mathematics and science tests (Wai et al., 2010; Lakin and Gambrell, 2014), concerns about raising young children (Williams and Ceci, 2012), and support from peers and family (Herzig, 2004). Collectively, these diverse challenges could present themselves at many points between earning a bachelor’s and Ph.D. degree, including choosing and then applying to graduate school, getting accepted, choosing the graduate school and mentor, completing coursework, developing research ideas and professional relationships, completing research projects, writing the Ph.D. thesis, and defending the thesis.

As described above, various factors at multiple time points could compel women to leave STEM fields at higher rates during the transition from the bachelor’s to the Ph.D. degree. However, empirically investigating such gender differences is methodologically challenging, especially because (1) few students pursue a Ph.D. after earning the bachelor’s and (2) the time in between the bachelor’s and Ph.D. can often exceed a decade (National Science Board [NSB], 2014). These challenges make prospective longitudinal studies exceptionally expensive, considering the large sample sizes and long time intervals needed.

Consequently, “[s]tudies of sex differences in Ph.D. completion are hampered by a lack of data” (Ceci et al., 2014, p.99). For instance, few research studies have systematically investigated gender differences in Ph.D. completion using representative samples (though see Xie and Killewald, 2012 for persistence rates ∼1–2 years after the bachelor’s). Prior studies have instead used students from self-selected fellowship programs (Bowen and Rudenstine, 1992; Myers and Pavel, 2011) or non-representative groups of institutions (Zwick, 1991; Council of Graduate Schools, 2008; Ampaw and Jaeger, 2011).

Other studies (Ceci et al., 2014; Gillen and Tanenbaum, 2014) have used population-level data to compare artificial cohorts of bachelor’s and Ph.D. degree earners (e.g., compare the percentage of women among physics bachelor’s degree earners in a given year and then among physics Ph.D. earners 8 years after). However, these studies make somewhat restrictive assumptions about the artificial cohorts. For example, these methods assume that students do not switch fields between the bachelor’s and Ph.D. degree and that students take similar amounts of time between the bachelor’s and Ph.D. degree. Hence, results even from these population-based studies could be strengthened and extended with alternate methods.

To help overcome these prior limitations, we used nationally representative samples and *retrospective* methods to investigate gender differences in the bachelor’s to Ph.D. STEM pipeline in the U.S. since the 1970s. As with all retrospective studies, the relevant events (e.g., earning of bachelor’s and Ph.D. degrees) had already occurred at the time of the survey; participants simply recalled their prior educational histories. This retrospective design allowed us to investigate changes in STEM persistence over three decades – a unique advantage of a retrospective, compared to prospective, longitudinal design.

Supplementing these retrospective analyses, cross-sectional analyses investigated how gender differences in three other characteristics (career goals, employment status, and family outcomes) varied across cohorts of bachelor’s degree holders. These supplemental analyses helped provide clues about why bachelor’s to Ph.D. persistence rates might have changed over time.

## MATERIALS AND METHODS

### OVERVIEW

For this study, the term *STEM persistence rate* refers to the percentage of students who earned a Ph.D. in a particular STEM field (e.g., engineering) among students who had earlier received bachelor’s degrees in that same field. We estimated persistence rates separately by field of study (e.g., engineering vs. physical science), bachelor’s degree cohort (e.g., 1980s vs. 1990s), and gender. These rates were estimated by two sets of numbers: (1) numbers of students who earned a bachelor’s degree in a particular field during a certain time frame and (2) numbers of those students who also later earned a Ph.D. in that same field. We used two national probability samples to estimate these two sets of numbers: National Survey of College Graduates (NSCG) and Survey of Doctoral Recipients (SDR).

### SAMPLES

The 2010 NSCG sample (*n* = 77,188) provided estimates for numbers of bachelor’s degree earners. The NSCG’s target population was college graduates living in the U.S. in 2010 under 76 years old who were not institutionalized (Fecso et al., 2012). The 2010 SDR sample (*n* = 31,462) provided estimates for numbers of Ph.D. earners. The SDR’s target population was a subpopulation of NSCG’s target population who also earned a Ph.D. from a U.S. institution in a science, engineering, or health field^3^. Although the NSCG sample could have also provided estimates for numbers of Ph.D. earners, the SDR sample provided more precise estimates given its exclusive focus on Ph.D. earners.

### ANALYZED VARIABLES

In both the NSCG and SDR surveys, participants were asked to recall their educational histories (e.g., the field of study and year of their first bachelor’s degree). Although retrospective studies such as ours can often have various recall biases (e.g., students misremembering how interested they were in science as children), it is unlikely that participants systematically misremembered concrete details such as what year they earned their first bachelor’s degree. These educational histories, participants’ demographics, and probability survey weights formed the basis for our analyses. Officials at the National Science Foundation created the survey weights to adjust for unequal sampling probabilities and non-response bias (Finamore et al., 2011). All the variables analyzed were available in the public-use versions of the 2010 NSCG and SDR surveys, which can be downloaded from the National Science Foundation’s website^4^. Lists of the analyzed variables and R analysis scripts are available in the supplemental materials for this paper.

### DEFINITION OF STEM FIELDS

We separated the category of “STEM” into five major subcategories as defined by the National Science Foundation’s classification system: (1) computer and mathematical science, (2) engineering, (3) life science, (4) physical science, (5), and social science (National Science Board [NSB], 2014). We also estimated persistence rates for pSTEM fields as a collective whole (categories #1, #2, and #4), given the focus on these fields in prior research on gender diversity in STEM (e.g., Riegle-Crumb et al., 2012).

### ESTIMATING PERSISTENCE RATES

We divided participants into cohorts based on field of study and year of the first bachelor’s degree (e.g., individuals who earned their first bachelor’s degree in engineering during 1976–1980). The NSCG sample provided estimates on the size of these cohorts. The SDR sample provided estimates on the numbers of Ph.D. holders within these cohorts (e.g., individuals who earned their first bachelor’s degree in engineering during 1976–1980 and who also earned an engineering Ph.D. before 2010). For any particular cohort, the persistence rate was estimated by dividing the sum of relevant SDR survey weights (i.e., the number of Ph.D. holders) by the sum of corresponding NSCG survey weights (i.e., the number of bachelor’s degree holders).

Analyses were restricted to U.S. citizens; the high proportion of international students among U.S. Ph.D. earners, but not bachelor’s degree earners, could have artificially inflated estimates of persistence rates (Xie and Killewald, 2012). Analyses included cohorts of students who earned their first bachelor’s degree between the years 1971–2000. These cohorts were divided into 5-year intervals (e.g., 1971–1975, 1976–1980, etc.) to increase sample sizes for individual cohorts and thus reduce fluctuations due to noise. See **Table 1** for sample sizes.

**Table 1:** Sample sizes by cohort, field of bachelor’s degree, and gender.

	Field of bachelor’s degree				
Cohort	Engineering	Life science	Math/computer science	Physical science	Social science
**National Survey of College Graduates**
′71–5	1073	543	275	347	1034
	40	271	146	109	825
′71–75	1116	584	250	407	839
	127	469	162	117	851
′81–85	1553	426	373	468	653
	284	376	239	186	777
′86–90	1483	323	564	306	695
	292	377	330	167	886
′91–95	1361	411	439	264	782
	329	440	275	135	1285
′95–00	1197	487	487	250	731
	338	659	270	195	1296
**Survey of Doctorate Recipients**
′71–75	297	482	137	492	516
	18	212	61	87	346
′71–75	361	538	154	475	395
	34	315	47	135	341
′81–85	584	423	159	559	309
	94	291	63	195	355
′86–90	497	377	174	406	314
	120	338	62	189	407
′91–95	392	396	151	378	331
	132	390	90	182	558
′95–00	259	420	106	303	254
	120	425	66	180	428

*Blue entries refer to men, and red entries refer to women.*

*Source: 2010 National Survey of College Graduates and 2010 Survey of Doctoral Recipients.*

### ESTIMATING STANDARD ERRORS

The 2010 NSCG survey used a complex two-phase sampling design in which individuals for the NSCG were sampled from respondents to the 2009 American Community Survey. As such, traditional approaches for estimating SEs in survey research (e.g., analytical formulas, jackknife replicates) are no longer appropriate. We therefore contacted the National Center for Science and Engineering Statistics and obtained custom SEs for this study’s specific estimates. These SEs were estimated using successive difference replication, which is appropriate for such two-phase sampling designs (White, 2014; Opsomer et al., under review). The sample design for the SDR survey was less complex and we therefore used standard “equivalent sample size” formulas to derive SEs for the SDR estimates (Potthoff et al., 1992).

### ACCOUNTING FOR THE LENGTH OF TIME BETWEEN DEGREES

By restricting analyses to cohorts in the year 2000 and before, we allow for at least 10 years between when students earned their first bachelor’s degree and when the surveys were conducted in 2010. Nevertheless, estimates especially for the last cohort (1996–2000) should be interpreted somewhat cautiously because a non-trivial proportion of students may earn Ph.D.’s after 2010.

The time in between the first bachelor’s degree and Ph.D. degree can be long. For instance, among U.S. citizens earning pSTEM Ph.D.’s in the U.S. between 2000 and 2010, the time in between degrees exceeded 10 years in 26% of cases, 15 years in 11% of cases, and 20 years in 7% of cases^5^. Given this long time between degrees, bachelor’s to Ph.D. persistence rates were likely somewhat underestimated especially among later cohorts (e.g., those who earned first bachelor’s degree in 1996–2000).

For these reasons above, we conducted additional analyses that compared persistence rates across cohorts based on the same length of time after the first bachelor’s degree. For instance, we compared persistence rates for the 1986–1990 cohort based on Ph.D.’s earned by 2000 with the persistence rates for the 1996–2000 cohort based on Ph.D.’s earned by 2010. Such additional analyses effectively control for the confound between cohort and length of time after the bachelor’s degree.

### ACCOUNTING FOR SAMPLE RESTRICTIONS

In both the NSCG and SDR samples, the target populations were restricted to non-institutionalized individuals living in the U.S. in 2010 aged 75 years old or younger. These restrictions on the target populations likely had only modest effects on our estimates. For instance, the restriction to non-institutionalized populations likely had little influence because of low incarceration rates among college-educated populations (Lochner and Moretti, 2004). The age restriction might have modestly influenced estimates especially for the oldest cohort (1971–1975). The age restriction, for instance, would have excluded individuals who earned their first bachelor’s degree in 1971 past the age of 36 years. However, few students in the U.S. earn bachelor’s degrees past the age of 36 years. For instance, only 3% of pSTEM bachelor’s degrees in 1993 were awarded to students older than 36 years^6^. Finally, the restriction to individuals living in the U.S. likely had small effects on our estimates because few U.S. bachelor’s degree earners move outside the U.S. after college graduation. For instance, less than 1% of pSTEM bachelor’s degree holders in 1993 moved outside the U.S. by the year 2003^5^.

### INTERACTIVE WEBSITE

We made an interactive website^7^ of our results to help interested readers inspect the effects of alternate analytic decisions (e.g., effects of using an alternate grouping of STEM fields or including non-U.S. citizens). All code to make this interactive website is also available in the supplemental materials.

## RESULTS

### RESULTS FROM RETROSPECTIVE METHODS

**FIGURE 1:**
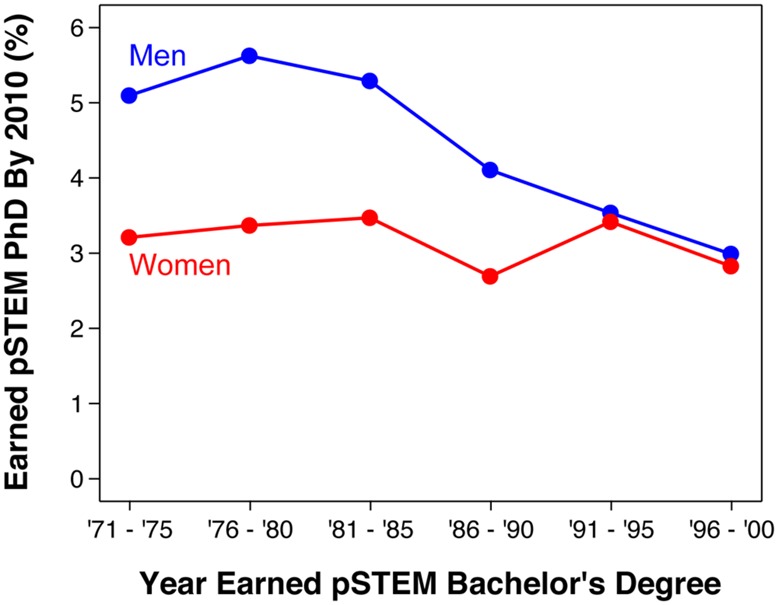
**Bachelor’s to Ph.D. STEM persistence rates by gender and bachelor’s degree cohort (excluding life and social science).** Rates especially for the last cohort (1996–2000) should be interpreted cautiously because a non-trivial proportion of students may earn Ph.D.’s in the future. Source: 2010 National Survey of College Graduates and 2010 Survey of Doctoral Recipients.

Among students earning pSTEM bachelor’s degrees in the 1970s and 1980s, women were 0.6–0.7 times as likely as men to later earn a pSTEM Ph.D. (**Figure 1**). However, this gender difference closed in the 1990s. Gender differences in persistence rates were statistically significant for cohorts in the 1970s and 1980s (all *p*s < 0.0005), but not in the 1990s (both *p*s > 0.60). See **Table 2** for count estimates that were used for calculation of persistence rates and SE for gender differences in persistence rates.

**Table 2:** Count estimates and gender differences in persistence rates for pSTEM fields.

	Count estimates						
	Bachelor’s		Ph.D.		Persistence rates		
Cohort	Female	Male	Female	Male	Difference	SE	*p*
’71–’75	77450	454251	2477	23098	1.89	0.51	**<0.001**
’76–’80	114323	448251	3838	25163	2.26	0.46	**<0.001**
’81–’85	185007	646401	6401	34129	1.82	0.37	**<0.001**
’86–’90	238176	635401	6385	26020	1.41	0.30	**<0.001**
’91–’95	174975	541446	5960	19085	0.12	0.34	0.727
’96–’00	196555	472214	5535	14065	0.16	0.31	0.604

*“Difference” refers to the percentage point difference in men’s minus women’s persistence rate. *p*s < 0.05 are bolded.*

**FIGURE 2:**
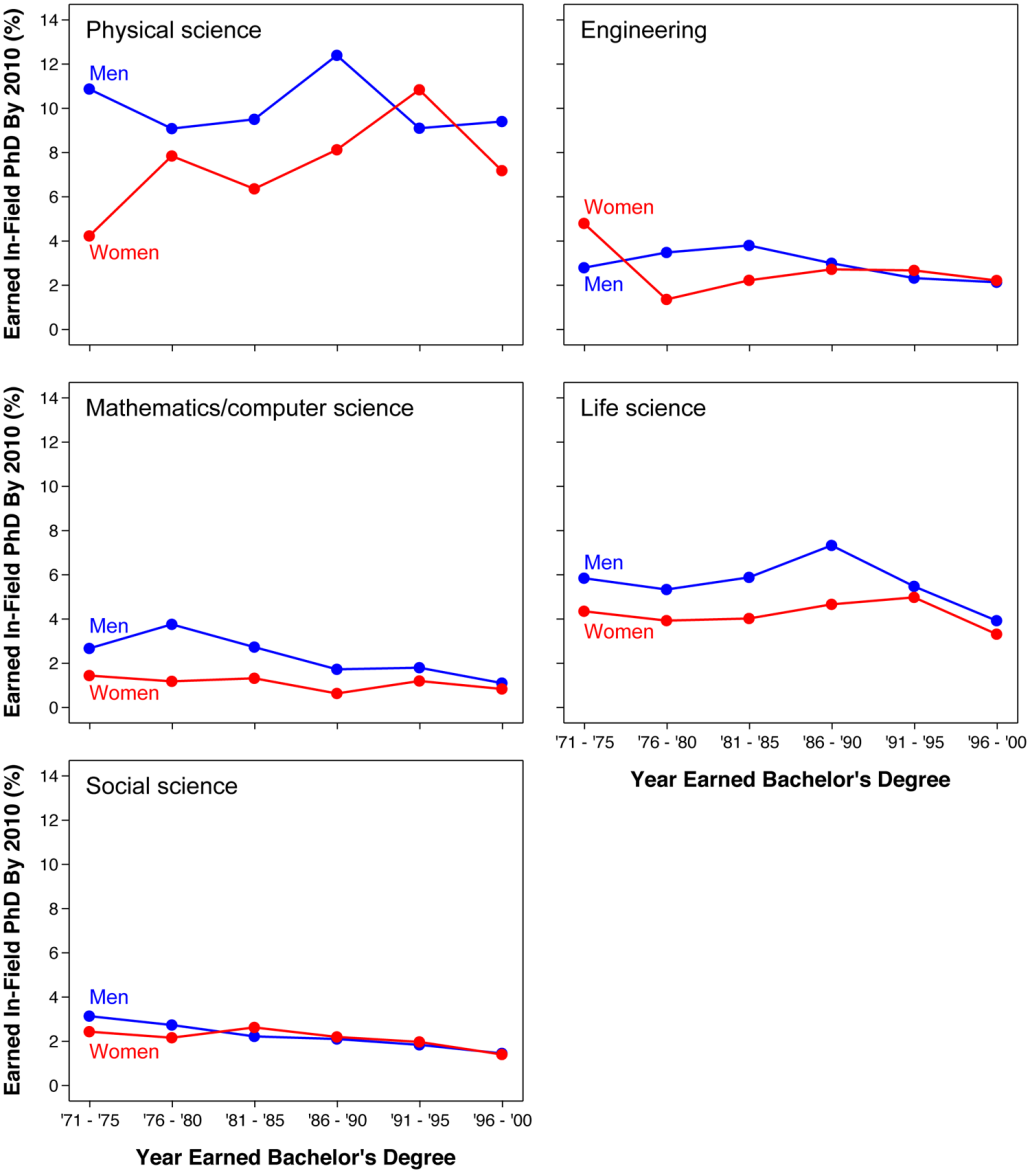
**Disaggregated bachelor’s to Ph.D. STEM persistence rates by gender and bachelor’s degree cohort.** Rates especially for the last cohort (1996–2000) should be interpreted cautiously because a non-trivial proportion of students may earn Ph.D.’s in the future. Source: 2010 National Survey of College Graduates and 2010 Survey of Doctoral Recipients.

As shown in **Figure 2**, similar results were found when disaggregating pSTEM fields (engineering, mathematics/computer science, physical science). Life science also showed a similar recent convergence between men and women. Social science had male advantages in persistence rates among cohorts in the 1970s, small non-significant female advantages in the early 1980s, and little to no gender differences since the late 1980s. Reasons for these convergences among cohorts in the 1990s varied across disciplinary fields (e.g., sometimes the convergence was driven by declines in men’s rates or increases in women’s rates, or both). No gender difference in persistence rates was significant for these 1990s cohorts (all *p*s > 0.19, except *p* = 0.054 for the 1991–1995 mathematics/computer science cohort). See Supplementary Table 1 for count estimates and SE across disaggregated fields.

**FIGURE 3:**
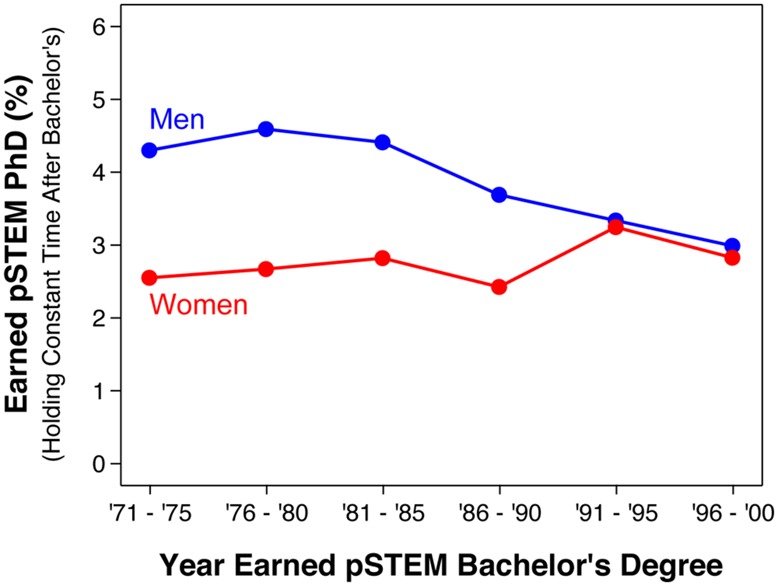
**Bachelor’s to Ph.D. STEM persistence rates by gender and bachelor’s degree cohort awarded (excluding life and social science), holding constant the length of time after the first bachelor’s degree.** For instance, rates for the 1996–2000 cohort were based on Ph.D.s earned by 2010, rates for the 1991–1995 cohort were based on Ph.D.s earned by 2005, rates for the 1986–1990 cohort were based on Ph.D.s earned by 2000, and so on. Source: 2010 National Survey of College Graduates and 2010 Survey of Doctoral Recipients.

As discussed earlier (see Accounting for the Length of Time between Degrees), one concern about these results was that cohort was confounded with the length of time after the bachelor’s degree. The analyses shown in **Figure 3** controlled for this confound by comparing pSTEM persistence rates over time using the same length of time after the bachelor’s degree. As shown, results were qualitatively similar compared to **Figure 1**: male advantages in pSTEM persistence rates were found in earlier cohorts in the 1970s, but not in later cohorts in the 1990s. Results were similarly unchanged for the groupings of STEM fields shown in **Figure 2**; see the interactive website for detailed results^7^.

### RESULTS FROM CROSS-COHORT COMPARISONS

**FIGURE 4:**
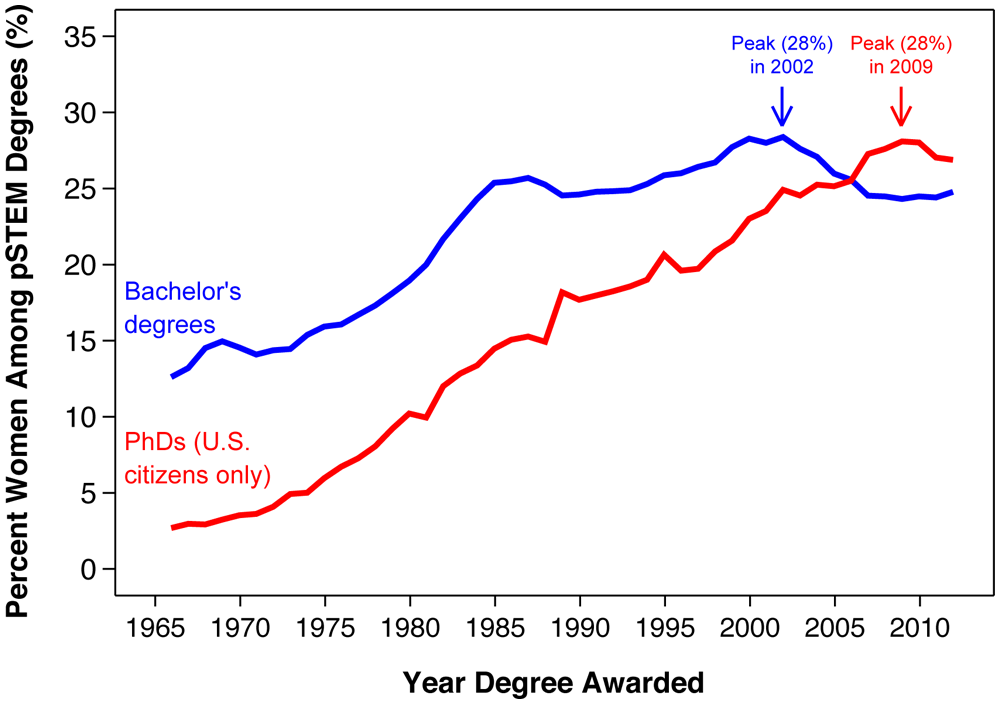
**Women’s representation among STEM bachelor’s and Ph.D. degree earners by year of degree awarded (excluding life and social science).** Ph.D. data after 2012 are not available. Source: WebCASPAR Integrated Science and Engineering Resource Data System (2014).

The results for persistence rates help to explain the continual increases in women’s representation among STEM Ph.D. holders. As shown in **Figure 4**, women earned less than 3% of the U.S.’s pSTEM Ph.D.’s in 1966, but earned 27% of them in 2012. This increase in women’s representation at the Ph.D. level has been steady over these four decades, and qualitatively similar trends are found across all STEM disciplines (e.g., life science and physics; Ceci et al., 2014).

Our results indicate that changes over time at the Ph.D. level can be attributed to two major factors: (1) the increase of women’s representation at the bachelor’s level among cohorts in the early 1970s to mid 1980s (**Figure 4**), and (2) the narrowing of gender differences in persistence rates among bachelor’s degree cohorts in the early 1980–1990s (**Figures 1 and 2**).

Although women’s representation among pSTEM Ph.D. holders has been continually increasing since the 1970s, this trend may not continue in the future for two major reasons: (1) women’s representation among STEM bachelor’s degree holders has been declining since 2000 (**Figure 4**), and (2) gender differences in STEM persistence rates have already closed (**Figures 1 and 2**). Current data indicate that women’s representation at the Ph.D. level has started to decline for the first time in over 40 years. The percent women among pSTEM Ph.D.’s awarded to U.S. citizens peaked at 28% in 2009 and has been declining ever since (**Figure 4**). Women would need to overtake men in bachelor’s to Ph.D. STEM persistence rates to reverse this trend. Otherwise, this trend will likely continue over the next few years.

### CHANGES IN OTHER CHARACTERISTICS AMONG BACHELOR’S DEGREE HOLDERS

To help place the bachelor’s to Ph.D. persistence findings in context, we investigated changes in other characteristics (e.g., career goals) for our focal bachelor’s degree holder population (i.e., U.S. citizens who earned a pSTEM bachelor’s degree during the years 1971–2000). These supplemental analyses used the NSCG data to characterize this focal population. Results revealed some stable gender differences regarding career goals (e.g., men were more likely to rate *salary* as a very important factor when thinking about a job, and women were more likely to rate *contribution to society* as very important) and employment outcomes (e.g., men were more likely than women to be working in 2010, working women were more likely than working men to be precollege teachers) in this focal population. However, these gender differences generally showed no consistent increase or decrease across cohorts; see the interactive website for complete, detailed results. Hence, gender differences in these characteristics likely cannot explain the cross-cohort changes observed for bachelor’s to Ph.D. persistence rates. Gender differences in having children and getting married were small across the cohorts and therefore also likely cannot explain the changes in persistence rates.

## DISCUSSION

The leaky pipeline metaphor has partially explained historical gender differences in the U.S., but it no longer describes current gender differences in the bachelor’s to Ph.D. transition in STEM. Remarkably, these recent convergences in persistence rates were found in all major groups of STEM fields (i.e., engineering, life science, mathematics, and computer science, social science, and physical science). These results align with and extend other recent studies that used alternate methods to investigate the bachelor’s to Ph.D. transition (Ceci et al., 2014; Gillen and Tanenbaum, 2014). Our study helps to place these recent convergences in historical context; some of the mixed results in prior literature likely reflect genuine change over time (e.g., Zwick, 1991; Herzig, 2004; Council of Graduate Schools, 2008; Gillen and Tanenbaum, 2014). Male Ph.D. holders still outnumber female Ph.D. holders by approximately three to one in pSTEM fields. However, our results indicate that gender differences in bachelor’s to Ph.D. persistence rates no longer help to explain this male overrepresentation. In fact, women’s representation in pSTEM is now *higher* at the Ph.D. than bachelor’s level.

Reasons for the convergences in persistence rates remain unclear. Sometimes the convergence was driven by declines in men’s rates (e.g., in mathematics/computer science), increases in women’s rates (e.g., in physical science), or both (e.g., in engineering). Our results helped eliminate potential hypotheses for these changes over time. For instance, convergences in persistence rates were likely unrelated to changes in some characteristics among bachelor’s degree holders. For instance, among pSTEM bachelor’s degree holders, gender differences in career goals and employment outcomes generally showed no consistent increase or decrease across the relevant cohorts. To explore other hypotheses, future research should investigate how changes in doctoral education might help account for the changes in persistence rates. For instance, gender diversity initiatives at the graduate level might have helped increase women’s rate of persisting in a doctoral program after entering graduate school.

### THEORETICAL IMPLICATIONS: GENERAL

Regardless of reasons why persistence rates might have changed over time, the recent convergences between women’s and men’s rates inform theories about women’s current representation in STEM. The convergences in rates may seem surprising given the multitude of factors that could cause women to leave STEM fields at higher rates than men (e.g., gender discrimination, gender-science stereotypes, right tail differences in cognitive abilities, or a combination of multiple factors). As reviewed in the introduction, many theories of women’s underrepresentation in STEM have often either explicitly or implicitly assumed that women are less likely than men to persist and pursue doctoral training in STEM. However, our results indicate that this foundational assumption may have been accurate in the past, but is no longer accurate.

One possible interpretation of recent gender similarity is that some factors could create male advantages in persistence rates (e.g., factors such as discrimination, right tail ability differences), but other factors create female advantages. For instance, self-selection among STEM undergraduates might create female advantages at the graduate level. As Hunt (2012, p. 1) hypothesized, various obstacles that female STEM undergraduates may face could “cause women entering science and engineering to be more positively selected for interest and aptitude than their male counterparts.” In other words, given the obstacles for female STEM undergraduates, only women with the strongest interest and aptitude for STEM would successfully earn STEM bachelor’s degrees.

This self-selection hypothesis, however, does not seem to align with the changes over time that we found. If anything, obstacles facing female STEM undergraduates were likely more extreme earlier in time when fewer women were earning STEM bachelor’s degrees (Ceci et al., 2014) and gender-science stereotypes were stronger (Miller et al., under review). According to this hypothesis, self-selection among female STEM undergraduates might have then been stronger in the 1970s and 1980s, meaning that those women might have been especially likely to pursue doctoral education. However, our results contradict this prediction because male advantages in persistence rates were larger earlier in time.

Another possible interpretation of these results is that various factors such as gender discrimination may not contribute substantially to current gender differences in bachelor’s to Ph.D. STEM persistence rates. In the following section, we consider this possibility for two specific factors especially relevant to doctoral education: gender discrimination among academic mentors and right tail differences in cognitive abilities. Of course, these possibilities are not necessarily mutually exclusive with the one discussed earlier (i.e., some factors such as self-selection create female advantages in persistence rates).

### THEORETICAL IMPLICATIONS: GENDER DISCRIMINATION

Two recent field experiments found that STEM faculty’s biases favor male students on average (Moss-Racusin et al., 2012; Milkman et al., 2014). For instance, in one nationally representative sample, STEM faculty ignored emails more frequently from prospective female graduate students than prospective male graduate students (Milkman et al., 2014). Such biases might therefore create a “leaky pipeline” for female STEM college majors by discouraging them from applying to graduate school or impeding their academic progress once in graduate school. However, our results do not agree with this basic prediction. Men and women now persist at roughly equal rates in STEM fields between the bachelor’s and Ph.D. degree, despite evidence of pro-male biases among academic mentors (Moss-Racusin et al., 2012; Milkman et al., 2014).

One possibility is that STEM faculty’s biases favor male students on average, but women overcome these biases by persisting at equal rates compared to men. Some empirical evidence supports this hypothesis. For instance, Milkman et al.’s (2014) study found biases favoring White males in nearly all academic fields. However, the size of the gender discriminatory gap in a particular academic field did not predict the representation of women in that field at the Ph.D. or faculty level. For instance, compared to non-STEM faculty, STEM faculty were not particularly biased against women. In fact, gender discrimination against White females was stronger among faculty in health fields than in the male-dominated fields of computer science and engineering (Milkman et al., 2014, **Figure 1B**). These results demonstrate that stronger pro-male biases do not necessarily translate to a lower representation of women at the Ph.D. or faculty level (see Ceci et al., 2014 for discussion of other related studies about gender discrimination in academic science).

These considerations above should not be used to discount the crucial importance of accurately assessing and changing gender biases in science. Gender discrimination can negatively affect many potential outcomes other than the numeric percentage of women in STEM fields. For instance, gender discrimination may cause some women to not feel respected or limit women’s equitable access to resources (e.g., equal salaries). Such realizations raise the question of whether diversity initiatives in STEM should focus more on increasing the representation of particular groups (e.g., women, non-Asian racial minorities) or improving the quality of experiences for members of those groups.

### THEORETICAL IMPLICATIONS: COGNITIVE ABILITIES

Some scholars have proposed that gender differences in mathematics and science reasoning performance might partially contribute to the underrepresentation of women in academic science (Benbow, 1988; Wai et al., 2010; Ceci et al., 2014). Although males and females often perform similarly on standardized mathematics tests on average, males are overrepresented in the right tail of mathematics and science reasoning performance (e.g., top 5% of performance or higher; Wai et al., 2010; Miller and Halpern, 2014). These right tail differences could be related to women’s representation among STEM Ph.D. holders because such individuals disproportionately come from this right tail of performance (Lubinski and Benbow, 2006) and individual differences in SAT-Mathematics scores at age 12 predict later differences in earning STEM Ph.D.’s even within the top 1% of performance (Wai et al., 2005).

Although these right tail differences could be relevant to women’s representation at the Ph.D. level, they are likely less relevant to representation at the bachelor’s level. Many students successfully earn STEM bachelor’s degrees without being in the right tail of mathematics performance (though see Hsu and Schombert, 2010 for additional discussion). For instance, only one-fifth (18%) of STEM bachelor’s degree holders in 2008^8^ had received a SAT-Mathematics score above 700^9^ in high school. Right tail differences in mathematics performance therefore likely do not substantially contribute to women’s representation in STEM fields at the bachelor’s level; longitudinal studies support this hypothesis (Riegle-Crumb et al., 2012).

If extremely high mathematics performance is required at the Ph.D. but not bachelor’s level, right tail differences in performance might be especially important for persisting from the bachelor’s to Ph.D. degree. For instance, low scores on challenging gatekeeper tests (e.g., GRE-Mathematics) could directly reduce students’ likelihood of being admitted to a STEM Ph.D. program. Hence, if right tail gender differences contribute to women’s representation among STEM Ph.D. holders, one might predict these right tail differences do so through their influence on persisting from the bachelor’s to Ph.D. degree.

Our results, however, do not agree with this basic prediction because men and women now persist at equal rates from the bachelor’s to Ph.D. in various STEM disciplines. Moreover, in pSTEM fields, men and women also persist at equal rates in the academic pipeline past the Ph.D. (see Ceci et al., 2014 for a review). For instance, in physical science and engineering fields, female and male Ph.D. holders are equally likely to earn assistant professorships (National Research Council [NRC], 2010) and academic tenure (Ginther and Kahn, 2009; Kaminski and Geisler, 2012; National Research Council [NRC], 2010). Hence, despite males outnumbering females in the top fraction of math and science reasoning performance (Wai et al., 2010; Miller and Halpern, 2014), males and females now persist at equal rates in the most intellectually challenging segments of the academic pipeline (e.g., earning Ph.D.’s and academic tenure) in some of the most math-intensive STEM fields (e.g., physical science and engineering). These results suggest women’s underrepresentation among high mathematics performers might be a more minor factor contributing to women’s underrepresentation among pSTEM Ph.D. holders and faculty.

### WHAT IS THE RIGHT METAPHOR?

Our research shows that the leaky pipeline metaphor is a dated description of gender differences in the transition between earning bachelor’s and Ph.D. degrees in STEM in the U.S. Related prior research indicates that the pipeline metaphor is also misleading for some other academic pathways in STEM. For instance, the metaphor fails to acknowledge the multiple entry points into STEM prior to the bachelor’s degree. Many students successfully earn STEM bachelor’s degrees despite not having traveled the traditional STEM “pipeline.” In one nationally representative study, 39% of STEM bachelor’s degree earners had not intended to enter STEM when asked in either 8th or 12th grade of secondary education (Cannady et al., 2014). Moreover, in another nationally representative sample, female science and engineering majors were likely to have joined STEM for the first time during college than have entered college already intending to major in STEM (Xie and Shauman, 2003). As Cannady et al. (2014, pp. 447–448) argued, such results make “the pipeline an ill-suited frame to understand STEM identity formation, particularly for women and underrepresented minorities.”

Nevertheless, the pipeline metaphor may be an apt description of academic transitions after the Ph.D. Academic pathways are considerably more rigid after the Ph.D. degree than before the bachelor’s degree. For instance, transitioning from a humanities Ph.D. to physical science tenure-track position would be nearly impossible without a physical science Ph.D.; the analogous transition between high school and college would be relatively open. However, as reviewed earlier, the post-Ph.D. academic pipeline leaks more women than men only in some STEM fields such as life science, but surprisingly not the more male-dominated fields of physical science and engineering (Ceci et al., 2014).

**FIGURE 5:**
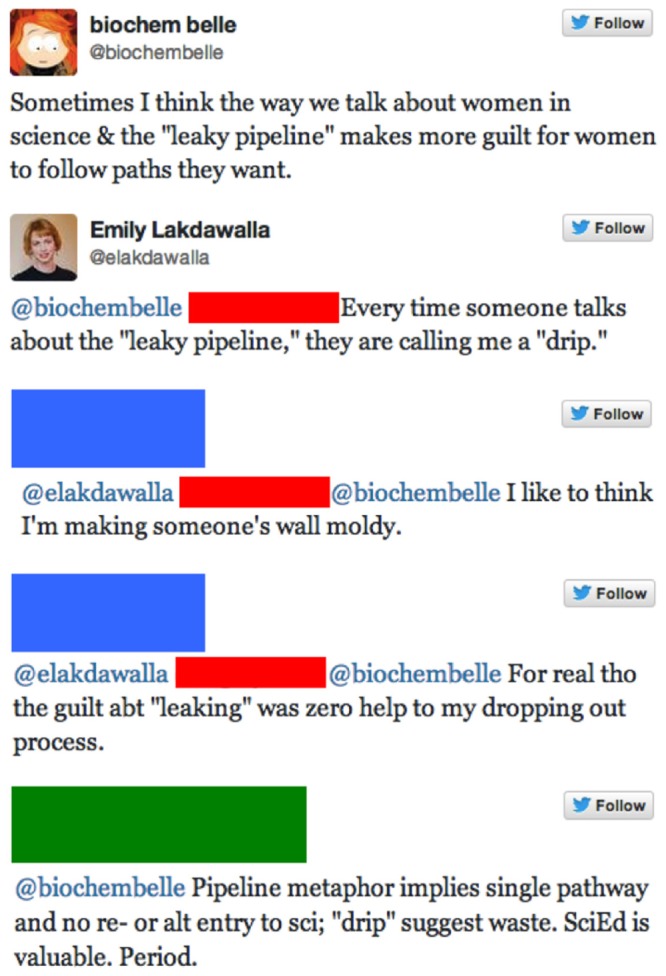
**Screenshots from a Twitter conversation regarding the “leaky STEM pipeline,” initiated by user *biochembelle* on 26 August 2013.** For further discussion, see http://biochembelle.com/2013/08/28/the-pipeline-isnt-leaky/. Twitter usernames are shown only for users who gave explicit permission.

Although the leaky pipeline metaphor may aptly describe the post-Ph.D. pathways in life science, the metaphor as a whole may nevertheless do more harm than good. It is an inappropriate description for nearly all other academic pathways in STEM. Moreover, the metaphor may even burden some women who leave academic science with a sense of guilt about being “leaks” in the pipeline. The Twitter user *biochembelle* wrote that, “Sometimes I think the way we talk about women in science and the ‘leaky pipeline’ makes more guilt for women to follow paths they want” (post on 26 August 2013). This sentiment resonated with other users who replied with tweets such as, “Every time someone talks about the ‘leaky pipeline,’ they are calling me a ‘drip”’ (user *elakdawalla*, tweet also on 26 August 2013). See **Figure 5** for other selected responses or the associated blog post by *biochembelle* for additional discussion^10^. These examples are of course anecdotal, but help illustrate how some individuals are personally impacted by the metaphor.

Along with other researchers (e.g., Xie and Shauman, 2003; Cannady et al., 2014), we propose replacing the metaphor of a singular pipeline with a network of multiple pathways into and out of STEM. This concept of pathways more accurately describes the multiple entry points into STEM prior to the bachelor’s degree. The idea also more positively portrays women who leave academic science as women pursuing other potentially fulfilling goals outside of academia (Webb et al., 2002; Xie and Killewald, 2012). And perhaps most importantly, this reconceptualization provides policy makers and educators with a wider range of strategies for increasing diversity in STEM. For instance, compared to “plugging the leaky pipeline” for female STEM undergraduates, equalizing gender differences in rates of joining STEM from non-STEM fields would more potently increase women’s representation among STEM bachelor’s degrees (Miller et al., under review).

### LIMITATIONS

The retrospective methods we used extended and complemented prior methods for studying gender differences in bachelor’s to Ph.D. persistence rates (e.g., Ceci et al., 2014; Gillen and Tanenbaum, 2014). However, the use of retrospective methods also limited our inferences to a subpopulation of degree earners who were included in the surveys’ target populations: non-institutionalized adults aged 75 years or younger living in the U.S. in 2010. However, as discussed earlier (see Accounting for Sample Restrictions), this limitation likely did not introduce large biases into our results. Our conclusions were also restricted to the U.S., though the retrospective methods that we used could be applied to any other nation with appropriate data.

Cohort was confounded with the length of time after the bachelor’s degree (see Accounting for the Length of Time between Degrees). As such, estimated persistence rates may have been modestly underestimated especially among the later cohorts; a non-trivial proportion of those students may earn Ph.D.’s after when the surveys were conducted in 2010. Importantly, however, our cross-cohort results were qualitatively similar when holding constant the length of time after the bachelor’s degree (e.g., see **Figure 3**). Hence, this limitation cannot account for the changes in gender differences over time. Future changes in persistence rates are unclear. Gender gaps could reemerge in the future, although our data offer no particular indication that they will reemerge.

Our methods revealed changes in persistence rates over time, but not in other outcomes relevant to doctoral education (e.g., performance in graduate school, subjective experiences of students). As we discussed earlier, future research should investigate whether some factors such as gender discrimination affect these other outcomes without substantially affecting persistence rates. Finally, continuing to investigate *why* persistence rates changed over time would also be invaluable.

## CONCLUSION

Overall, these results and supporting literature point to the need to understand gender differences at the bachelor’s level and below to understand women’s representation in STEM at the Ph.D. level and above. Women’s representation in computer science, engineering, and physical science (pSTEM) fields has been decreasing at the bachelor’s level during the past decade. Our analyses indicate that women’s representation at the Ph.D. level is starting to follow suit by declining for the first time in over 40 years (**Figure 2**). This recent decline may also cause women’s gains at the assistant professor level and beyond to also slow down or reverse in the next few years. Fortunately, however, pathways for entering STEM are considerably diverse at the bachelor’s level and below. For instance, our prior research indicates that undergraduates who join STEM from a non-STEM field can substantially help the U.S. meet needs for more well-trained STEM graduates (Miller et al., under review). Addressing gender differences at the bachelor’s level could have potent effects at the Ph.D. level, especially now that women and men are equally likely to later earn STEM Ph.D.’s after the bachelor’s.

## Conflict of Interest Statement

The authors declare that the research was conducted in the absence of any commercial or financial relationships that could be construed as a potential conflict of interest.

## Footnotes

1 http://ncsesdata.nsf.gov/webcaspar/

2 Prior research on gender diversity in STEM has often focused on these pSTEM fields because women are especially underrepresented in them (Ceci et al., 2014).

3 http://www.nsf.gov/statistics/srvydoctoratework/

4 http://sestat.nsf.gov/datadownload/

5 These estimates were calculated from the NSCG sample. Variables to compute the exact number of years between the first bachelor’s degree and Ph.D. degree were not available in the public-use SDR dataset.

6 These estimates were based on our own analysis of the 1993 Baccalaureate and Beyond study, which can be analyzed on the PowerStats website for the National Center for Educational Statistics (http://nces.ed.gov/datalab/powerstats/).

7 http://d-miller.shinyapps.io/bachelorsPHD

8 These estimates were based on our own analysis of the 2008 Baccalaureate and Beyond study, which can be analyzed on the PowerStats website for the National Center for Educational Statistics (http://nces.ed.gov/datalab/powerstats/).

9 SAT-Mathematics scores of 700+ corresponded to the top ∼5–10% of performance among the SAT test-taking population. See http://professionals.collegeboard.com/profdownload/sat_percentile_ranks_2008_males_females_total_group_math.pdf

10 http://biochembelle.com/2013/08/28/the-pipeline-isnt-leaky/
