# Supplementary material for: The bachelor’s to Ph.D. STEM pipeline no longer leaks more women than men: a 30-year analysis
Source: Front Psychol. 2015 Feb 17;6:37. doi: 10.3389/fpsyg.2015.00037 (PMC4331608; doi:10.3389/fpsyg.2015.00037)
Supplement: Supplementary file 1 [file 122519_Miller_Table_1.DOCX]

**Supplementary Table 1. Count estimates and gender differences in persistence rates across disaggregated fields.** “Difference” refers to the percentage point difference in men’s minus women’s persistence rate. *p*s < .05 are bolded.

|  | | | Count estimates | | | | | |  | | | | |
| --- | --- | --- | --- | --- | --- | --- | --- | --- | --- | --- | --- | --- | --- |
|  |  | | Bachelor’s | |  | | PhD | |  | | Persistence rates | | |
| Cohort | | Female | | Male | | Female | | Male | | Difference | | SE | *p* |
| **Engineering** | | | | | | | | | | | | | |
| '71 - '75 | | 7894 | | 259758 | | 377 | | 7186 | | -2.00 | | 1.68 | 0.233 |
| '76 - '80 | | 35004 | | 251064 | | 467 | | 8675 | | 2.12 | | 0.45 | **<0.001** |
| '81 - '85 | | 66392 | | 381692 | | 1459 | | 14405 | | 1.58 | | 0.41 | **<0.001** |
| '86 - '90 | | 74110 | | 373263 | | 1996 | | 11084 | | 0.28 | | 0.45 | 0.540 |
| '91 - '95 | | 68683 | | 316348 | | 1817 | | 7268 | | -0.35 | | 0.44 | 0.429 |
| '96 - '00 | | 71053 | | 239120 | | 1556 | | 5044 | | -0.08 | | 0.39 | 0.834 |
| '96 - '00 | | 7894 | | 259758 | | 377 | | 7186 | | -2.00 | | 1.68 | 0.233 |
| **Life science** | | | | | | | | | | | | | |
| '71 - '75 | | 107940 | | 204189 | | 4670 | | 11886 | | 1.49 | | 0.70 | **0.033** |
| '76 - '80 | | 165607 | | 231899 | | 6463 | | 12313 | | 1.41 | | 0.54 | **0.009** |
| '81 - '85 | | 143362 | | 170375 | | 5733 | | 9981 | | 1.86 | | 0.70 | **0.008** |
| '86 - '90 | | 141374 | | 119691 | | 6557 | | 8742 | | 2.67 | | 0.91 | **0.003** |
| '91 - '95 | | 144468 | | 155332 | | 7158 | | 8467 | | 0.50 | | 0.69 | 0.470 |
| '96 - '00 | | 229643 | | 211984 | | 7555 | | 8268 | | 0.61 | | 0.48 | 0.199 |
| **Mathematics/computer science** | | | | | | | | | | | | | |
| '71 - '75 | | 43521 | | 101959 | | 617 | | 2697 | | 1.23 | | 0.52 | **0.018** |
| '76 - '80 | | 52291 | | 88266 | | 605 | | 3296 | | 2.58 | | 0.58 | **<0.001** |
| '81 - '85 | | 67204 | | 138064 | | 870 | | 3735 | | 1.41 | | 0.41 | **0.001** |
| '86 - '90 | | 125327 | | 191645 | | 762 | | 3259 | | 1.09 | | 0.23 | **<0.001** |
| '91 - '95 | | 83325 | | 147645 | | 977 | | 2619 | | 0.60 | | 0.31 | 0.054 |
| '96 - '00 | | 86625 | | 173636 | | 704 | | 1872 | | 0.27 | | 0.24 | 0.275 |
| **Physical science** | | | | | | | | | | | | | |
| '71 - '75 | | 26035 | | 92534 | | 1094 | | 10036 | | 6.64 | | 1.41 | **<0.001** |
| '76 - '80 | | 27028 | | 108921 | | 2113 | | 9866 | | 1.24 | | 1.71 | 0.470 |
| '81 - '85 | | 51410 | | 126645 | | 3256 | | 12003 | | 3.14 | | 1.26 | **0.013** |
| '86 - '90 | | 38740 | | 70493 | | 3138 | | 8720 | | 4.27 | | 1.97 | **0.031** |
| '91 - '95 | | 22967 | | 77453 | | 2483 | | 7029 | | -1.74 | | 2.12 | 0.413 |
| '96 - '00 | | 38877 | | 59458 | | 2783 | | 5576 | | 2.22 | | 1.94 | 0.251 |
| **Social science** | | | | | | | | | | | | | |
| '71 - '75 | | 350849 | | 445207 | | 8451 | | 13855 | | 0.70 | | 0.27 | **0.009** |
| '76 - '80 | | 375920 | | 364185 | | 8028 | | 9870 | | 0.57 | | 0.27 | **0.030** |
| '81 - '85 | | 327338 | | 331129 | | 8513 | | 7270 | | -0.40 | | 0.27 | 0.129 |
| '86 - '90 | | 405851 | | 341067 | | 8814 | | 7083 | | -0.09 | | 0.24 | 0.698 |
| '91 - '95 | | 583272 | | 383904 | | 11349 | | 6960 | | -0.13 | | 0.19 | 0.478 |
| '96 - '00 | | 568150 | | 345756 | | 7822 | | 4933 | | 0.05 | | 0.17 | 0.772 |
