# Supplementary material for: The bachelor’s to Ph.D. STEM pipeline no longer leaks more women than men: a 30-year analysis
Source: Front Psychol. 2015 Feb 17;6:37. doi: 10.3389/fpsyg.2015.00037 (PMC4331608; doi:10.3389/fpsyg.2015.00037)
Supplement: Supplementary file 2 [file 122519__Data_Sheet_1.ZIP › AnalysisCode/bachelorsPHD/introduction.html]

## Explore Gender Differences in the Bachelor's to PhD STEM Pipeline

This interactive webpage accompanies the *Frontiers in Psychology* manuscript
"The Bachelor’s to PhD STEM Pipeline No Longer Leaks More Women Than Men: A 30-Year Analysis," written by David I. Miller and Jonathan Wai. We designed this webpage to help interested readers
inspect the effects of alternate analytic decisions (e.g., effects of using an alternate
grouping of STEM fields or including non-U.S. citizens).
  
  
Click the "Results" tab to begin.
The default settings will reproduce Figure 1 from the manuscript.

  
  

### Abstract

For three decades, research and public discourse about women’s underrepresentation in academic science have often focused on the “leaky pipeline” metaphor. According to this model, women are more likely than men to leave science at multiple points from the beginning of college through academic tenure. We used retrospective longitudinal methods to investigate how accurately this model has described the bachelor’s to PhD pipeline in science, technology, engineering, and mathematics (STEM) fields in the United States (U.S.) since the 1970s. Among STEM bachelor’s degree earners in the 1970s and 1980s, women were less likely than men to later earn a STEM PhD. However, this gender difference closed in the 1990s. Qualitatively similar trends were found across STEM disciplines. The leaky pipeline metaphor therefore partially explains historical gender differences in the U.S., but no longer describes current gender differences in the bachelor’s to PhD transition in STEM. Our results help constrain theories of how various factors contribute to women’s underrepresentation in STEM. Overall, these results point to the need to understand gender differences at the bachelor’s level and below to understand women’s representation in STEM at the PhD level and above.
